# Coronavirus as a Catalyst to Transform Consumer Policy and Enforcement

**DOI:** 10.1007/s10603-020-09462-0

**Published:** 2020-06-12

**Authors:** C. Riefa

**Affiliations:** Brunel Law School, Brunel University, London, Uxbridge UK

**Keywords:** Price gouging, Consumer law enforcement, Online platforms, Vulnerable consumers, Principle-based approach, Duty to trade fairly

## Abstract

A review of the unfair commercial practices (including price gouging) that have emerged in the context of the pandemic lead us to reflect on how effective consumer law enforcement is at this juncture. This article calls for the pandemic to act as a catalyst to review the way consumer law has so far approached markets and their regulation. It argues that now, more than ever, consumer law needs to protect the vulnerable and public enforcement mechanisms must be able to prevent harm as much as possible rather than repair it. Fairness should be by design and not something that is offered to consumers simply as a remedy. The article explores some viable solutions to effect this transformation of consumer policy and enforcement beyond the pandemic.

## Impact of the Pandemic on Consumer Rights

The coronavirus pandemic is having very severe consequences. Notwithstanding the devastating loss of life worldwide, all aspects of society have sustained dramatic changes. Many consumers may find themselves in precarious financial situations, unable to pay the bills and at risk of building arrears, following a loss of income or an increase in the price of living. Holidays and non-essential travel have been curtailed for the foreseeable future and will in any event be drastically altered even when lockdowns are lifted or relaxed.

Most of daily life has shifted online, at least for those who are fortunate enough not to be digitally excluded. Lockdown has also accelerated the growth of electronic commerce with online shopping being adopted by categories of shoppers who had so far resisted the migration, a trend set to continue post-pandemic. Those changes to consumer habits per se should not impact consumer rights. On the contrary, intuitively, one may expect that at a time where economies are in difficulty, ensuring an increased number of consumers spend the money they have ought to promote stronger competition. While some businesses have led the way in playing fair, many others have fallen short and tried to circumvent consumer rights. For example, companies cancelling weddings have still required payments, and airlines are issuing vouchers instead of refunds, a practice contrary to the law in the European Union (EU). Those practices have spread across the EU and further afield.

Figures on the exact scale of the problem are still sparse, but the figures from the Competition and Markets Authority (CMA) for the United Kingdom (UK) give a sense of the problem. The CMA reported having received 14,000 complaints through its online form (CMA 2020b). Out of those, cancellation complaints amount to 4 out of 5 complaints received, and were concentrated among a small number of large firms: a quarter of businesses account for 70% of complaints; the 3 most complained about businesses account for 17% of total complaints; the top 10 businesses account for 28% of the total. Price gouging (the practice of increasing prices to an unfair/exploitative level) also spread, notably on online platforms. Pasta, masks, hand sanitisers, and toilet rolls were offered for sale at extortionate prices. In the UK, price increases reported to the CMA showed that hand sanitiser prices have increased 367% for example, and rice, which was the lowest price increase reported, hovered around 50%. The median increase was 130% for complaint data concerning rice, eggs, toilet paper, flour, paracetamol, hand sanitisers, out of a total of 430 complaints (CMA 2020b). Unfortunately, the Covid-19 pandemic has also unleashed a range of scams that are the purview of criminal law enforcement. From quack cures to a range of phishing activities, scammers have capitalised on fear, and to some extent also greed, at this time of great uncertainty. They have struck on and off-line. Overall, levels of complaints for coronavirus-related practices have spiked. In the UK again, 21,000 complaints have been filed with the CMA as at 19 April (CMA 2020b) and over £2.7 million has been lost to fraudsters according to Action Fraud. The total loss experienced is difficult to gauge at this point, as any early data does not account for the hidden face of consumer detriment (which may include unreported crime, as well as loss of consumer surplus as a result of undetected price rises or when consumers will be unable to return goods bought online within the normal time frames for example). Nevertheless, it is clear that consumers, right across the EU and further afield, are suffering detriment that the application of consumer law ought to curtail and or at least repair. In many cases, however, it is not the case.

Of course, in EU countries, there is ample legislation to deal with all those unfair practices, and neighbouring disciplines, such as competition, data protection, or contract law, may also assist in some cases. However, while the pandemic does not change the existence of consumer rights per se, it has a disruptive effect in the way consumer rights can and will be exercised. The enforcement of consumer rights has become all the more challenging at a time where courts may have been shut down (if only momentarily) and many alternative dispute resolution (ADR) bodies are not equipped to fully migrate online, revealing the fragility of a legal system over-reliant on private actions to keep markets in check. Besides, the pandemic has also brought to the fore the limitations that public enforcers face in the protection of economic rights and the means at their disposal to combat poor practices that transcend geographical boundaries.

A review of the practices that have emerged and endure in the context of the pandemic demonstrates that consumer law enforcement is rather ineffective to meet the demands of consumers. This article calls for the pandemic to act as a catalyst to review the way consumer law has so far approached markets and their regulation. I argue that now, more than ever, consumer law needs to protect the vulnerable, and public enforcement mechanisms need to be able to prevent harm rather than repair it. Fairness should be by design (Siciliani et al. 2019, pp.187–209) and not something that is offered to consumers simply as a remedy.

## Inadequacy of Consumer Enforcement to Meet the Demand for Protection in Times of Pandemic and Beyond

Consumer law enforcement normally takes place via private and public enforcement mechanisms. Both are limited in their ability to protect consumers. The pandemic has not changed the nature of these issues. Consumer enforcement has always been notoriously difficult and somewhat under-funded. But the pandemic has magnified the problems and brought them to the fore for all to see. While refund problems or price gouging practices have always existed, they would normally be practices experienced by a fairly niche number of consumers at any one time. The scale of the pandemic has multiplied the amount of consumers concerned by those practices, and hit all sectors of activity at once, not just nationally but internationally as well. All consumers, rich or poor, are affected one way or another by those practices. In many ways, the pandemic has also exacerbated the vulnerabilities that may be experienced by consumers. This is, of course, problematic because, in the same way that the impact of Covid-19 on life is felt more acutely by the most disadvantaged in our societies, consumer rights and their lack of enforcement also unduly disadvantages more vulnerable consumers (Caplovitz 1967).

At this juncture, there is a need to acknowledge the shortcomings of enforcement models that were built around competition law as a main pillar (Cseres 2005) and consumer empowerment and private enforcement as a main vehicle for remedies. Indeed, historically, consumer law has often been seen as a poor parent of competition law. Competition and market forces are often billed as the best protectors of consumer interests (Ramsay 2012, p.3), and consumer law has tended to prefer information as a way to assist consumers rather than more substantial obligations for businesses, or active monitoring of markets by enforcers. Consumer law was built for the “homo economicus,” a consumer that is “reasonably well informed, reasonably observant, and circumspect” (*Gut Springenheide and Tusky* 1998) and thus is not easily misled. Although termed the “average consumer,” this standard is very much anchored on consumer self-reliance and bears little resemblance to what actual consumers do or think.

Besides, consumer enforcement by and large rests on private enforcement by consumers. In a time of pandemic, this enforcement method proves even more inadequate than in normal times as consumers find themselves preoccupied by other priorities (notably assuring an income), or because advice may not be as accessible as it would normally be. In any event, their first brush with consumer law enforcement will be with a system that is by and large already failing them.

At best of times, consumers often shy away from enforcing their rights. They may fail to do so for a variety of reasons: absence of knowledge that the right exists in the first place, complexity of the ADR landscape (Money Saving Expert 2017), delays, perceived difficulties in accessing courts, reduced or no legal aid for consumer cases, etc. The net result is that consumers using courts or ADR tend to be a narrow sample of the population. They are primarily middle-aged, middle-income men with high educational attainment (BEIS 2018). Besides, their use of the system may be even more difficult in situations where the purchase was cross-border. There, data from the European Consumer Centres Network (ECC-Net), which has been offering a valuable service to consumers in the EU for over 15 years, reveals a surge in queries. But, in the situations where consumers gain assistance, this will mostly be in the form of information from ECC-Net. It will take an average of 120 days for an intervention with a trader, and nearly half of the cases dealt with will not result in a satisfactory outcome for consumers, forcing them to explore other options (ECC-Net 2020a, b). With the timing of a refund critical to a consumer at a time of pandemic, this will hardly be satisfactory. Collective action mechanisms are also lacking in many EU countries, despite reforms being underway to introduce an EU wide collective action procedure. In the UK, for example, the collective action mechanisms (known as Group Litigation Orders) are primarily used for high-value disputes and not appropriate for aggregating a number of small-value economic losses. Besides, most consumer law enforcement activity is targeted at a single firm or a small group of firms. It thus struggles to address poor behaviours that may develop across a particular sector or industry. Anyhow, another key issue is that consumers are relying on their rights *ex-post*, i.e., after the damage has already occurred, leaving them to carry the financial burden of mistakes induced by unfair traders.

On the other side of the enforcement coin, enforcement of consumer law tends to rest on a system of public enforcement that, while effective in many respects, is very much limited, decreasing its ability to offer relief where and when it is most needed. Public enforcement remains mostly reactive rather than proactive, and focussed on a small number of traders who engage in unfair practices. Besides, enforcers tend to prioritise enforcement in areas where aggregate harm is most acutely felt. Some Member States may have at their disposal some tools such as market studies and investigations that can lead to interventions shaping markets before widespread detriment occurs. This is the case in the UK, in particular. Those tools are, however, not as widely used as they could.

Enforcers adopt different intervention styles, but enforcement of consumer law has a tendency to rest on a collaborative approach. For example, in the UK, the CMA chief executive urged retailers to “behave responsibly throughout the coronavirus outbreak and not to make misleading claims or charge vastly inflated prices”. He also reminded members of the public that these obligations may apply to them, too, if they resell goods, for example on online marketplaces (CMA 2020a). A Covid-19 taskforce was launched to tackle those negative effects of the crisis that fell within its remit. Many enforcers across the EU issued guidance on their expectations regarding cancellations and refunds, or price gouging and other unfair practices. In the UK, this simply resulted in the CMA monitoring practices and being concerned about firms not issuing refunds at all, making the refund process unjustifiably complex to discourage consumers, charging fees, or pressuring consumers to accept vouchers instead of refunds. No formal action has been taken at the time of writing, other than issuing statements to the effect that the CMA is prepared to take businesses who flaunt its guidance to court (CMA Guidance 2020). A similar stance was adopted regarding price gouging. The CMA wrote to industry and trade bodies setting expectations and seeking further information concerning price rises.

Many the companies had written to report that price rises further up the supply chain were causing the problem, an issue which the CMA were still trying to verify at the time of writing. This highlights the difficulty consumers would face in trying to pursue such matter on their own accord. Gathering the required evidence can be extremely challenging and consumers would seldom have the required resources to do so.

The CMA’s approach, which coincides with that of the European Commission, was to write to platforms asking for them to act. As a result, eBay, Amazon, and others, have introduced price gouging policies, and have committed to monitoring Covid-19 related unfair commercial practices on their sites. However, despite those measures being put in place and action being taken, many practices still escape detection and continue to cause consumer detriment. While the CMA has declared that it is ready to warn as well as to take stronger enforcement action where there is evidence that consumer or competition law has been broken, no action has yet been taken. This can be explained by the CMA being in a difficult situation, because it found that it does not hold the powers that are necessary to act to tackle price gouging.

In contrast, in Italy, AGCM, the enforcer, has access to enforcement tools much more focussed on the application of administrative powers (that the UK enforcer is lacking). It has intervened more strongly to close websites selling quack cures or stop search engines pointing to illegal pharmacies, for example. However, power to combat price gouging are also lacking in Italy, and the Autorita’ Garante della Concorrenza e del Mercato (AGCM) is only investigating practices for now. In contrast in France, the practice was controlled with the adoption of specific legislation fixing a price ceiling for the sale of hand sanitisers. However, both the approaches in Italy and France, although more heavy-handed than the UK’s, are still failing to detect many unfair practices, and are very much limited by the resources the enforcement bodies have. This means that most enforcers have a limited ability to repair the harm that consumers experienced in a timely manner. Also, they also often only have limited powers to recover money on behalf of consumers, meaning that consumers may be protected for the future, but the detriment already suffered is not always compensated.

## The Pandemic as a Catalyst to Transform Consumer Law Policy and Enforcement

The pandemic can and should be used as a catalyst for refrom. It can lead to reflections on the way consumer law has operated to date and to exploring the possibility of a change of direction.

The first aspect to mention is the need to preserve the sanctity of consumer protection over the economic interests of businesses when the going gets tough. This is, of course, a delicate question, but during the Covid-19 epidemic, the fragility of consumer rights was exposed. Admittedly, this point, needs to be put into a wider context going beyond the remit of this piece, but which consist in interrogating whether or not airlines (and other businesses) are justified in retaining payments and refusing refunds so they could weather the financial storm, but at the detriment of consumers. The speed at which the idea was floated in EU countries is indicative of how quickly economic rights can be downgraded in times of crisis, and how frail consumer protection may in fact be. A number of Member States are reported to have made requests to the European Commission to “suspend” the application of consumer rights in order for airlines to avoid giving refunds and forcing consumers to accept vouchers. The motion was backed by the majority of the Council.

My instinctive view is that consumer rights need to be upheld at all times, and not doing so may amount to an unfair commercial practice. This is the case in particular in situations where the businesses in question are supported by government bailout schemes and furlough schemes, as is the case in the UK and many other Member States. Refusing a refund to consumers who are entitled by law to receive one, requires consumers to effectively suffer a loss multiple times over: first, they will lose money they require at a time where they themselves are out of work or finding financing their household more challenging. Secondly, they may also loose the value in the future, as a rebooking may be plausible but the consumer may no longer be able to go, or incur further costs to enjoy the flight, holiday, or services when it is finally provided. At that point, they may lose the value with no recourse. In addition, if bailouts are offered to the industry in question, consumer will also indirectly contribute via taxation. This also needs to be considered against the backdrop of airlines still paying dividends to shareholders or receiving bailouts even though they are located in tax heaven and have not contributed taxes in the past. Consumers also have to carry the burden of claiming their rights in court or via ADR, facing yet again additional expenses in the process.

I am comforted to see that upholding consumers’ right to a refund is a view shared by other consumer advocates (e.g., BEUC 2020) and regulators. The European Commission has also taken a clear position on this (European Commission 2020). However, it is disappointing that allowances are made and a preference for vouchers is expressed when consumers ought to receive refund. The CMA in the UK explained that it is prepared to offer some flexibility as to the timing of the refund (CMA Guidance 2020), whereas the EU Commission has taken its position to make voucher schemes more attractive. This notably includes ensuring that vouchers can be redeemed for cash if they are not used during the period of validity.

The concern here is that not strongly upholding rights as they were originally devised erodes the rule of law, and it also erodes consumer confidence. This is particularly pertinent because traditionally, consumer law has been disconnected from the realities of the person it protects. There is an artificial separation between consumers and citizens (Davies 2016; Hesselink 2007) which prevents a coherent and inclusive system of protection. Seeing vouchers as a viable alternative (the Commission calls it a win-win) is denying consumers access to more basic social needs while they extend credit to the airlines and other providers.

While consumer law is now awaking to the needs of the vulnerable consumer, the concept “seems to encompass still rather superficially those who cannot, or can no longer, cope with the requirements of modern consumer society” (Reich 2016, p. 149), carrying a tangible risk of stigmatisation with it (O’Hara 2020, p.1). But the pandemic has showed that consumer protection ought to embrace the fact that as humans, we are all vulnerable, and this is a constant characteristic—a universal, ever-present experience, which may be exposed at any given moment by our individual circumstances or embeddedness (Fineman 2008, 2017; Riefa and Saintier 2020). In many respects, the pandemic reminds us that “vulnerability” needs to become a core value of consumer protection policies again. Consumer law was indeed developed out of social justice concerns in the 1960s and 1970s, and it is those same concerns that ought to lead once more, putting values protecting the weaker members of society at its core (Brown 2016; Domurath 2016; Twigg-Flesner et al. 2018; Willett 2018).

To do this, there is a need to rebalance the traditional relationship between competition and consumer law when it comes to enforcement. Developing a more equal footing for consumer law will allow resources to be

“best allocated to the enforcement authority most able to protect consumers and repair the harm caused. This is especially important in those cases where prompt intervention under consumer law can prevent the issue from deteriorating to the point where not even competition enforcement would suffice to restore a fair market outcome. In turn, more reliable consumer enforcement ought to help develop a general duty to trade fairly, shaping markets for the future and lessening the need for competition enforcement, that is, thanks to the fact that the presence of more confident and assertive consumers empowers competition on the merits.” (Siciliani et al. 2019, p.9).

One area where this could be very successful is in combatting Covid-19 related unfair practices such as price gouging. This is because price gouging is normally dealt with under specific price legislation (see, e.g., France) or via the application of competition law (exploitative abuses, under Art 102 TFEU). However, “cases of pure unfair pricing are rare in competition law. Authorities find them difficult to bring and are, rightly, wary of casting themselves in the role of price regulators” (Cary et al. 2020). Within the context of a pandemic, the task is even more arduous because the exploitative prices normally flow from the exploitation of market power, whereas during the pandemic, the ability to charge exploitative prices may not correlate with dominance. In those conditions, meeting the test developed in *United Brands* (1976) whereby a price is considered exploitative if it has no reasonable relation to economic value of the product supplied, would be particularly challenging.

However, controlling price gouging could be done by applying the Unfair Commercial Practices Directive 2005/29/EC (UCPD). Note that enforcement action could not occur as a misleading practice, as the price is normally clearly disclosed to consumers, and it would be unlikely to be seen as aggressive as consumers are not coerced in the purchase (other than the impression of shortages being created possibly even outside the remit of the retail site or platform on which the goods are up for sale). It could nevertheless be tackled by applying the “general clause” of the UCPD, because the practice is contrary to the requirements of professional diligence and materially distorts (or is likely to materially distort) the economic behavior of the average consumer^1^. The general clause has rarely been used by enforcers, because it has been perceived to be there only to close regulatory gaps rather than as the main tool (Micklitz 2009). The pandemic therefore appears to be precisely the time to use it. For enforcers, two main obstacles may loom. The first is the need to clear the hurdle of the standard of the average consumer. However, there is already leeway contained in the legislation itself in lowering the expectations the courts have placed on the average consumers (Siciliani et al. 2019, p.36). Besides, “to achieve a higher standard of consumer protection, the normative standard of professional diligence should discourage opportunism but promote trustworthiness and, more prescriptively, assistance. Accordingly, it could be argued that the material distortion is due to the trader’s implicit refusal to assist both potential and current customers to avoid making mistakes. Therefore, there would be no need for a complex inquiry into the conduct of competing traders in order to establish the appropriate benchmark of professional diligence” (Siciliani et al. 2019, p. 201).

However, if the use of consumer law can come to help plug some gaps in competition law enforcement, it cannot replace enforcers having access to adequate regulations and tools to take action.

In a UK context, for example, having adequate tools would mean holding sufficient administrative powers to take action rather than relying on the court’s intervention even for a violation of an undertaking already given by the business to stop or modify a practice, or requiring the enforcer normally to consult businesses before any enforcement action is started (s.214(4A) Enterprise Act 2002). At a more general level, it is about finding ways to ensure enforcers are responsive but also seek a more preventative role, possibly more akin to market surveillance models.

On the regulatory side, it has become essential to review the obligations placed on platforms and the remit they ought to have in acting themselves as regulators of behaviours. The pandemic has revealed how unfair practices not only spread online via platforms, but has also increased reliance of consumers on online providers. Platforms were already incredibly powerful and one of the most common interfaces for consumers in the digital world. The pandemic has further entrenched their position. Aside from the thorny issues of how competition law ought to be able to shape the digital market, the question of how much liability platforms should shoulder when transactions occurring or originating on their platform cause harm to consumers remains unsettled (Riefa 2016; Van Eecke 2011).

In the EU, the liability regime set out by the E-commerce Directive (ECD) provides an exemption from liability for illegal content and activities, in respect of which the platforms, acting as a mere conduit or host, do not have control or knowledge over, and upon obtaining such knowledge act expeditiously to disable access (Article 14 ECD). The European Court of Justice however has limited the scope of this protection. Immunity is only granted to neutral hosts that behave like diligent economic operators in their discovery and removal of any litigious materials (*Google v Louis Vuitton* 2010; *L’Oréal*^2011^). However, it is not possible to impose general monitoring obligations on online platforms under Article 15 ECD, although national legislation can impose specific obligations, such as ordering a host to remove identical and/or equivalent content previously declared illegal (Eva Glawischinig-Piesczek v Facebook 2019). The discourse on liability has somewhat shifted, and there is growing appetite for increasing the liability that should befall intermediaries, at least when it comes to secondary liability (Frosio 2017; Van Eecke 2011). The work leading up to a reform of the electronic commerce directive (known as the Digital Services Act) announced by Von der Leyen (2019) indicates some willingness to consider stricter liability including with a view to protect consumers on platforms (European Parliament 2020) although it seems to revolve around a strengthening of information duties (bar protection against unsafe goods and discussions regarding misleading online advertisement). It is my view that intermediaries ought to take on more liability especially when they benefit financially either directly (e.g., payment of a commission or fee) or indirectly (e.g., collection of personal data) for breaches of consumer protection rules (their own of course, but also third parties). This could be done in part by increasing the general monitoring that ought to take place to prevent consumer harm. Platforms also ought to be made to establish procedures enabling enforcers to have direct notice and take down processes in place to signal infringements to consumer laws (including notably, unfair commercial practices and unfair terms) as well as use of software wherever possible to help with detection (Micklitz et al. 2017; Riefa 2016, p.216). Going further, it should also be possible for consumers to report content as they are most likely to have first-hand knowledge of any potential detriment. Had those steps been in place already, removal of Covid-19 unfair commercial practices on platforms would likely have been much faster, reducing consumer detriment and removing the need for the European Commission and national enforcers to ask for assistance.

This leads to exploring the values of goal-based approaches (Decker 2018) where businesses are box ticking rather than truly concerned by consumer welfare and where all that is not forbidden is thus allowed. Moving towards performance-based consumer law (Willis 2015) and/or a more economic approach to consumer law policy and enforcement (Siciliani et al. 2019) could offer better ways of making consumer law robust and resilient in the future. What those approaches have in common is that they tend towards putting more emphasis on businesses behaving fairly as a general guiding principle, although they all see the role of information and disclosure in different ways. Willis (2015) called for a performance-based approach that would encompass a comprehension performance standard and a suitability standard. This call for change is motivated by the fact that rules on disclosures and product design, which focus on the actions of firms (rather than the effect those have on consumers) have failed. Firms have framed disclosures or reformulated products to evade product design rules and the regulation of transaction terms. Willis explains that “performance-based consumer law together with ongoing field-testing has the potential to incentivize firms to educate rather than obfuscate, to develop simple and intuitive product designs that align with, rather than defy, consumer expectations, and to channel consumers toward products that are suitable for consumers’ circumstances”. But Willis does not advocate supplanting disclosures and design regulation, but rather to supplement them at least in the foreseeable future. By contrast, Siciliani et al. (2019) are more critical of information as a means to effect consumer protection. They advocate the creation of a general and positive duty to trade fairly, which could be defined in flexible ways so as to reverse the burden that currently befall on the consumers to be the effective guardians of competition by voting with their feet. The way to do this is by allowing enforcers to work with some alternative economic models (away from behavioural economics and reconnecting with bounded rationality) to assess consumer detriment and use existing laws (albeit with some amendments) to prevent it. “A more economic approach to consumer policy making and enforcement allows to move beyond the dominant transparency paradigm to an approach where firms have a positive duty to treat consumers fairly and shape their commercial offers in a way that prevents consumers from making mistakes. Over time, this fairness by design approach will emerge as the only acceptable way to compete.”

The coronavirus pandemic is far from resolved and will likely continue to disrupt the exercise of consumer rights long into the future. However, it must act as a catalyst for action to commence now on the process of improvements that are needed to ensure consumer policy and enforcement remain fit for purpose. A review of the practices that have emerged and endure in the context of the pandemic have illustrated that consumer law enforcement is far from optimal and in need of an overhaul. Now, more than ever, consumer law needs to protect the vulnerable, and public enforcement mechanisms need to be able to prevent harm rather than repair it. Fairness should be by design (Siciliani et al. 2019, pp.187–209) and not something that is offered to consumers simply as a remedy.

## Footnotes

1 In the UK, a right of private action is offered under the CPRs, but only for misleading action or aggressive practices. In this sense, the general clause is incomplete and does not offer the highest standards of protection. This would inevitably need to be addressed, for private redress (which should include individual as well as collective actions) ought to come and complement public enforcement efforts.
